# A Comparative Study of *Diospyros malabarica* (Gaub) Extracts in Various Polarity-Dependent Solvents for Evaluation of Phytoconstituents and Biological Activities

**DOI:** 10.1155/2022/4746223

**Published:** 2022-06-25

**Authors:** Zohra Zreen, Amjad Hameed, Shumaila Kiran, Tahir Farooq, Mohammed Suleiman Zaroog

**Affiliations:** ^1^Department of Applied Chemistry, Government College University Faisalabad, Pakistan; ^2^Nuclear Institute for Agriculture and Biology (NIAB), P.O. Box: 128, Jhang Road Faisalabad, Pakistan; ^3^Department of Biochemistry, Faculty of Applied Medical Sciences, University of Gezira, Wad Medani, Sudan

## Abstract

Keeping in mind the ascribed repute of *Diospyros malabarica* (*D. malabarica*), this investigation was commenced to assess the effect of diverse solvents on extraction yields, phytochemical components and antioxidant capability, and *in vitro* biological activities of *D. malabarica* for pharmaceutically active constituents to combat various infections. To screen phytochemicals both qualitatively (flavonoids, terpenoid, saponins, tannins) and quantitatively like total phenolic and flavonoid contents, *Diospyros malabarica* parts include the following: root, leaves, bark, stem, ripe, and unripe fruit were sequentially extracted with organic solvents such as petroleum ether, dichloromethane, ethyl acetate, ethanol, methanol, and water in increasing order of polarity from less polar to more polar solvents. Furthermore, biological activities such as antibacterial, antifungal, anticancer, antidiabetic, and anti-inflammatory were explored. The results revealed that all the tested solvents displayed a vital role in the extraction yield, the content of phytochemicals, and the studied biological activities. Methanol was found as the best solvent followed by the ethanol for the extraction, representing the highest extraction yield (18.3%), rich diversity of phytochemicals, and the highest total phenolic contents (602 ± 0.001 *μ*g EAG/mg of extract) and total flavonoid contents (455 ± 0.6 *μ*g EQ/mg of extract) in bark extract. Furthermore, methanol bark extract showed high *in vitro* antibacterial activity (30.25 mm ± 0.9), antifungal activity (18.25 mm ± 0.2), anticancer activity (48%), antidiabetic activity (68%) and anti-inflammatory activity (62%) followed by ethanol amongst other extracts of *D. malabarica*. Accordingly, methanol might be as an ideal solvent to get maximum content of phytochemicals, promising antioxidants, and *in vitro* biological activities from bark extract amongst other extracts of *D. malabarica* compared to pet ether, ethyl acetate, and dichloromethane and may act as free radical rummager because phytochemical constituents exhibit antioxidant capability. Our findings suggest that phytochemical compounds (flavonoids, tannins, phenols, saponins, and terpenoids) found in the bark extract of *D. malabarica* may be attributed to evaluate potent anti-inflammatory, anticancer, antidiabetic, antibacterial, and antifungal activities.

## 1. Introduction

Herbal medicines and extracts are a rich source of unprocessed drugs with medicinal properties. According to the World Health Organization, 80% of the worldwide people uses diverse plant components and their active ingredients as traditional therapies [1–4]. Therapeutic plants have tremendous healing sound effects due to the presence of several biologically active compounds such as flavonoids, terpenoids, phenols, saponins, resins, and steroids [5]. Phytochemical investigations are underway to find out the pharmacologically active compounds from plants possessing secondary metabolites which provide a complete defense system and are the main factors of therapeutic efficacy for curing many chronic diseases [6]. Free radicals are formed during metabolic processes as necessary intermediates through diverse endogenous roots (breathing, mitochondria, incitement of granular leukocyte, peroxisomes and phagocytic cells, etc.) and exogenous causes (pollution, radiation, smoking, certain drugs, toxins, insecticides, and heavy metals and natural solvents) [7]. An increased number of free radicals and inadequate antioxidant defense system causes oxidative damage. To suppress the hazard of a variety of free radicals and ROS, potent antioxidants possessing the capability to rummage free radicals by neutralizing them are required [8]. Though both natural (plant-based) and man-made (chemically synthesized) antioxidants are helpful to promote health and protect the biological system by suppressing free radicals, plant-based antioxidants are reported best as they inhibit the formation of free radicals without any side effects [9, 10].

Many studies stated that man-made antioxidants as butylated hydroxyanisole (BHA) and butylated hydroxytoluene (BHT) have toxic effects on human health through lipid, protein, and DNA damaging [11]. The harmful effects of these artificial antioxidants have required the exploration of new natural products having antioxidant characteristics. Recent investigations indicated that the plant-based antioxidants have been known to shield humans from a few prolonged ailments, for example, aggravation, immune system sicknesses, malignancy, and tumor arrangement by preventive the production of free radicals [12]. The research on plants has been increasing worldwide, and scientific data collected so far reflects the use of medicinal plants in many traditional systems [13].

*D. malabarica*, “Gaub plant,” is an evergreen tree with ornamental usage in Pakistan, well adopted in this climate and producing flowers and fruits. It is one of the indigenous medicinal plants granted with strong antioxidant activity [14]. The various parts of *Diospyros kaki L.* (Ebenaceae) like leaves and fruit have historically being used to treat high blood pressure and atherosclerosis owe to their antiproliferative and anti-inflammatory attributes [15, 16]. As a rich source of pharmacologically active bioconstituents, *D. malabarica* could be anticipated for its use as a phytomedicine. It has been reported previously that all organs of this plant, especially fruits, bark, and leaves, are used in medicinal preparation due to its antioxidant potential as possessing potency to combat various disorders in many traditional medicinal systems of the world [14]. Various extraction techniques with different optimization conditions [17] for natural products have been given in Table 1.

Moreover, the leave extracts are used for the treatment of burning, diabetes, atherosclerosis, intermittent fever, and cancer and reflect potent antidiabetic [18] antimicrobial, anti-inflammatory [1], and antipyretic activities [19]. The alcoholic extract of the stem revealed the anticancer and antidiarrheal activities. The methanol extract of *D. malabarica* ripe fruits has shown antibacterial, antitumor, antioxidant, hepatoprotective, antidiabetic, and antidiarrhoeal activities [20, 21]. Hypoglycemic and antihyperglycemic, antibacterial, and antiurolithiatic activities [22] were as follows. The traditional uses of *D. malabarica* have been reported as continued onset in reduction of gastrointestinal motility, inhibition of prostaglandin synthesis, and diarrhea [23]. Subsequently, extensive investigations on diverse organs of *D. malabarica* regarding comparative phytochemical and biological investigations have not been executed up till now.

In this study, we prepared different extracts of *D. malabarica* organs using organic solvents in order of increasing polarity from less to more polar solvents to extract both polar and nonpolar phytoconstituents. The phytochemicals of extracts were explored both qualitatively and quantitatively for biological activities. These extracts were further studied for their *in vitro* anticancer, anti-inflammatory, antibacterial, antifungal, and antidiabetic properties to figure out the most significant and ideal plant part as a potent future source of herbal drugs by virtue of its antioxidant competency.

## 2. Experimental Data

### 2.1. Sample Collection

Different organs including young fresh green leaves, unripe fruit and stem, dark black bark, and fully ripe fruit of only one sample of *D. malabarica*, “Gaab plant,” were collected from the plant nursery of Nuclear Institute for Agriculture and Biology (NIAB), Faisalabad, Pakistan, and were identified by the botanist. From July 2017 to Sep. 2017, plastic baskets saved in labeled containers in freezer at 10°C to maintain the integrity were carried to the laboratory for investigation. The samples were cleaned with water to get rid of dust and then rinsed with deionized water for analysis. The analysis was conducted at NIAB (MAB Lab-1), Faisalabad, Pakistan.

### 2.2. Drying and Grinding

The selected and carefully washed plant organs (leaves, bark, and stem, ripe and unripe fruit) were air-dried for 72 hours at 25°C to 30°C. The dried samples were then finely grounded by mortar and pestle into a powder and kept in labeled clean airtight bottles at room temperature for further analysis.

### 2.3. Preparation of Extracts

To extract various polarity-based chemical constituents, the extracts using different solvents were prepared by sequential extraction from less polar solvents to more polar solvents [24]. 50 g of powdered plant organs was dissolved in 500 mL solvent and petroleum ether (40-60°C) and kept in an airtight container. After that, it was supported to keep for 72 hours at room temperature having continuous shaking until soluble material was broken down to form a solution. Whatman filter paper No. 42 was used to filter extract. The marc left after extraction was air-dried and again extracted with solvent, dichloromethane for another 72 hours. This was followed by the extraction with ethyl acetate, ethanol, methanol and finally, water. The five extracts of each organ of *D. malabarica* were well-found. After complete evaporation of solvent, all extracts were solubilized in 10% dimethyl sulphoxide (DMSO) to get final concentration of 50 mg/mL and kept at 5°C in sanitized covered labelled bottles till further experimentation [25].

### 2.4. Extraction Yield

**Figure 1:**
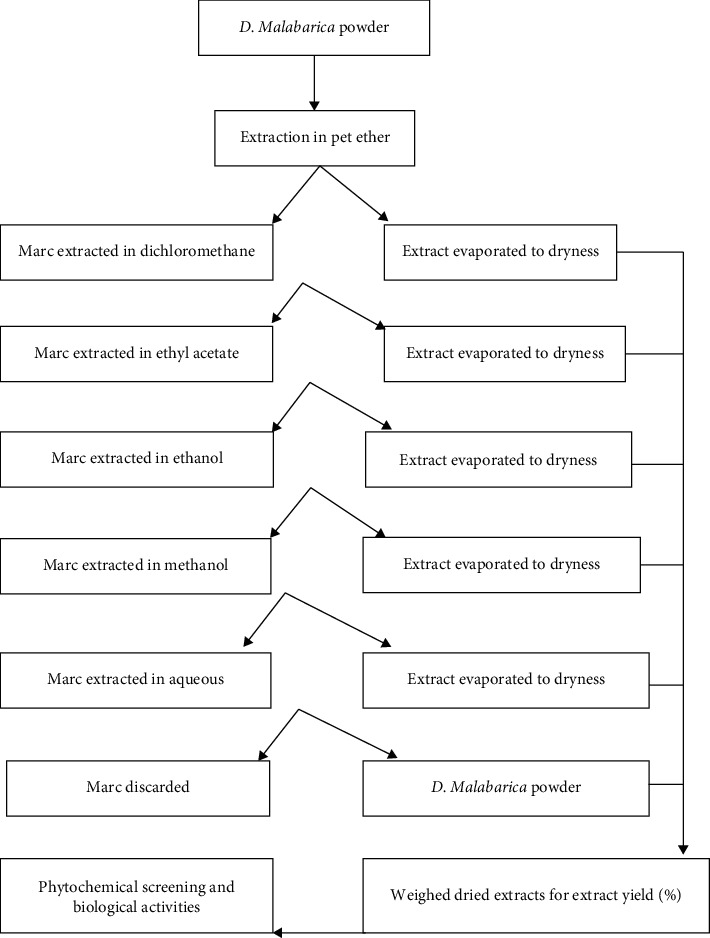
Schematic representation for extraction of phytochemicals.

The ratio between the obtained mass of the dry plant extract and the total mass of plant material processed in the experiment are called the performance of crude extract [26]. This yield can be calculated with the help of the following formula (Figure 1):

(1) $$\begin{array}{c} \%\text{Yield}=\text{weight of extract}/\text{weight of dry powder}\times 100. \end{array}$$

### 2.5. Phytochemical Screening

The phytochemical screening was conducted on the basis of precipitation reactions or coloring. According to the Houghton and Raman method [27], plant organs were converted directly into powder form. Phytochemical analyses along with antibacterial and antifungal activities were conducted in the Applied Chemistry Hi-Tech Laboratory of Govt. College University, Faisalabad, Pakistan. Likewise, biological activities such as anti-inflammatory and antidiabetic activities were executed at plant breeding and genetics division (MAB Lab-1) in Nuclear Institute for Agriculture and Biology (NIAB), Faisalabad, Pakistan while anticancer activity was investigated at Hussain Ebrahim Jamal Research Institute of Chemistry, Karachi, Pakistan.

#### 2.5.1. Qualitative Screening of Phytochemicals of Extracts

Following standard protocols were executed in order to find out the presence of tannins, terpenoids, saponins, flavonoids, and total phenol and flavonoid contents [28].

*(1) Tannins*. 500 mg of each dry sample was heated in 5 mL of H_2_O. Then, after filtration few drops of ferric chloride (FeCl_3_) solution was mixed, the development of bluish-black color confirmed the tannin [28].

*(2) Saponins*. 200 mg of the powdered sample was boiled in 500 mL deionized H_2_O and filtered. Then, 10 mL of filtrate and 5 mL of deionized H_2_O were mixed and agitated extensively to get a strong constant lather. Then, the lather was mixed with olive oil (3 drops) and blended extensively until the formation of emulsion [28].

*(3) Flavonoids*. 5 mL of 1.0 M diluted NH_3_ solution was mixed in 10 mL of each filtered aqueous sample, and then 5 to 6 drops of concentrated H_2_SO_4_ were added to this solution. The resulted yellow color was an indication of flavonoids [28].

*(4) Terpenoids*. 5 mL plant sample and 2 mL of CHCl_3_ were mixed vigorously. After that, 3 mL of concentrated sulphuric acid was added with care to get a layer. The appearance of the reddish-brown color on the inner face was a sign of terpenoids [29].

#### 2.5.2. Quantitative Screening of Phytochemicals

*(1) Test for Total Flavonoid Contents (TFC)*. Following the established colorimetric method with few modifications, the TFC was estimated in different extracts of *D. malabarica* using quercetin as standard described by Hossain et al. (2019). 5 mg of each plant extract was added in 4 mL of methanol in volumetric flask (5 mL). After this, 4 mL of distilled H_2_O was added in 1 mL of each plant sample. To this reaction solution, 0.15 mL of 10% NaNO_3_ was added. After five minutes, 0.15 mL of 10% AlCl_3_ was added. Then, this mixture was incubated for six minutes followed by the addition of 1 mL of NaOH. At the same time, different concentrations (450, 400, 350, 300, 250, 200, 150, 100, and 50 *μ*g/mL) of quercetin as standard were prepared in the same practice as defined in sample extracts. Then, after incubation, the absorbance of sample and standard solutions was recorded against methanol blank at 510 nm by UV-visible spectrophotometer. Total flavonoid contents of all tested plant extracts were determined by calibration curve of quercetin standard, and the attained results of TFC were expressed as quercetin equivalents (*μ*g quercetin/mg dry sample). All the estimation for TFC investigation in the extracts was done in triplicates [30].

*(2) Total Phenolics Contents (TPC)*. For the estimation of phenolic compounds in different plant extracts of *D. malabarica*, an established FCR (Folin-Ciocalteau (*F*-*C*) reagent) method was followed with few modifications by using gallic acid as standard stated by Al-Saeedi et al. (2016) [31]. For evaluation, an ice-cold pestle and mortar were used to homogenize 5 mg each plant sample in 0.5 mL ice-cold methanol (95%) in volumetric flask of 5 mL. Then, for incubation, plant samples were kept for 48 hours at ambient temperature in darkness. At room temperature, the plant samples were rotated at 14,462 × g for 5 minutes. The clear liquid that lies above was then separated for TPC analysis. At first, 100 *μ*L supernatant and 10% (v/v) Folin–Ciocalteu reagent (FCR) (100 *μ*L) were mixed and vortexed vigorously, and 700 mM sodium carbonate (800 *μ*L) was taken in test tubes and incubated for 1 hour at lab temperature. At 765 nm, the absorbance of blank corrected sample solutions was measured. With the help of different gallic acid concentrations (450, 400, 350, 300, 250, 200, 150, 100, and 50 *μ*g/mL), a standard curve was drawn, and a linear regression equation was measured. By using the linear regression equation, phenolic contents of samples equivalent to gallic acid were determined (*μ*g EAG/mg of the dried sample) [31].

### 2.6. Biological Activities

#### 2.6.1. Anti-Inflammatory Activity (In Vitro Inhibition of Albumin Denaturation)

The inhibition technique of albumin denaturation was pursued to evaluate anti-inflammatory action. Method of [32] was used to execute tests with few modifications. 10 mg of diclofenac sodium was used as a standard drug. An aqueous solution of 1% bovine albumin serum was prepared and adjusted at pH 6 using 1 M HCl. The reaction solution was involving test extracts at a concentration of 5 mg/mL of 10% DMSO to obtain stock solutions. These reaction mixtures were further used to produce two final concentrations of 100 and 400 *μ*g/mL of 10% DMSO of test substances to find plant activity at low and high concentration. 450 *μ*L of 1% BSA was added to 50 *μ*L of test extract, and its volume was increased to three times. Then, all the sample solutions and standards were incubated for 20 min. at 25°C and then heated at 70°C in a water bath for 5 minutes to denature the protein. The turbidity was measured using a spectrophotometer after cooling the reaction mixtures at 660 nm. Three concordant readings were taken [32]. % inhibition of protein denaturation was calculated as follows:

(2) $$\begin{array}{c} \%\text{inhibition}=\frac{Abs\text{ control}-\text{Abs sample}}{\text{Abs control}}\times 100, \end{array}$$

where Abs_control_ is the absorbance without sample, and Abs _sample_ is the absorbance of plant extract/standard.

#### 2.6.2. Anticancer Activity

For the evaluation of anticancer action of composites in 96-well level-bottomed microplates, standard 3-[4, 5-dimethylthiazole-2-yl]-2, 5-diphenyl-tetrazolium bromide (MTT) colorimetric measure was utilized [33]. Minimal Essential Medium Eagle was utilized as the culture for HeLa cells, enhanced with 5% of fetal bovine serum (FBS), 100 IU/mL of penicillin, and 100 *μ*g/mL of streptomycin in flasks (75 cm^2^) and kept in 5% carbon dioxide incubator at 37°C. Then, harvested exponentially developing cells were tallied with a hemocytometer and diluted with a specific [34] medium. The cell culture was prepared with a concentration of 6 × 10^4^ cells/mL and brought into 96-well plates (100 *μ*L/well). After keeping on incubation the whole night, the medium was detached, and a fresh medium of 200 *μ*L was added having (1-30 *μ*M) different concentrations of compounds. After two days, 200 *μ*L of MTT (0.5 mg/mL) was added to wells and again incubated for four hours. Afterward, dimethyl sulfoxide (100 *μ*L) was added to all wells. The plant extract was prepared at concentration of 100 *μ*g/mL in DMSO (1%). Doxorubicin as standard drug was also prepared in the same way and was also utilized as positive control. The extent of MTT reduction to formazan in cells was recorded by taking absorbance at 570 nm, utilizing *μ*plate per user (Spectra Max Plus, Molecular Devices, CA, and USA). Anticancer activity was calculated as fixation causing development hindrance of half (IC_50_) for HeLa cell lines. The % inhibition was determined with the following formula:

(3) $$\begin{array}{c} \%\text{inhibition}=\frac{{\text{Abs}}_{\text{control}}-{\text{Abs}}_{\text{sample}}}{{\text{Abs}}_{\text{control}}}\times 100. \end{array}$$

%inhibition = the results was organized by using SoftMax Pro software (Molecular Device, USA).

#### 2.6.3. Antidiabetic Activity (In Vitro *α*-Amylase Inhibitory Activity)

The 3,5-dinitrosalicylic acid (DNSA) method was followed to assay *α*-amylase inhibition [35] with few modifications. 5 mg of each plant extract of *D. malabarica* was dissolved in a minimum amount of 10% DMSO and was further dissolved in 20 mM sodium phosphate buffer and 6 mM NaCl at pH 6.9 to prepare two concentrations of 100 and 400 *μ*g/mL. The reaction mixture was consisting of 200 *μ*L *α*-amylase solution (2 units/mL) with 200 *μ*L of the plant extract and was allowed to stand in an incubator at 30°C for ten minutes. Then, 200 *μ*L of 1% starch solution in distilled H_2_O (w/v) was added in all extracts and incubated for three minutes. 200 *μ*L DNSA reagents (12 g of Rochelle salt in 8 mL of 2 M NaOH and 20 mL of 0.096 M of 3, 5 DNSA solutions) were further added to terminate the reaction and then boiled in a water bath for ten minutes at 90°C. After cooling all the reaction mixtures were diluted with the addition of 5 mL distilled H_2_O, the absorbance was measured by UV-visible spectrophotometer at 540 nm. 200 *μ*L of buffer was used as a substitute for plant extract to prepare blank with 100% enzyme activity. Another blank reaction was also prepared in the absence of the enzyme solution at each concentration using the plant extract. In the same way, positive control was prepared by using acarbose (100 *μ*g/mL–2 *μ*g/mL) as a standard drug, and reaction was performed similarly to the reaction with plant extract as mentioned above.

The *α*-amylase inhibition was calculated as % inhibition using the equation as follows:

(4) $$\begin{array}{c} \%\text{inhibition}=\frac{{\text{Abs}}_{\text{control}}\;\left(100\%\right)-{\text{Abs}}_{\text{sample}}}{{\text{Abs}}_{\text{control}}\left(100\%\right)}\times 100. \end{array}$$

### 2.7. Antibacterial Activity

#### 2.7.1. Bacterial Strains

The antibacterial activity of plant extracts was assessed by using two bacterial strains*, Escherichia coli* (MG1655), the gram-negative bacteria, and *Streptococcus* (ATCC25925), the gram-positive bacteria. The bacterial strains were provided from the culture collection of Botany Dept. Government College University Faisalabad, Pakistan.

*(1) Preparation of Inoculum and Test Solutions*. The bacterial strains were separately subcultured for 24 hours at 37°C in nutrient agar pates to get well-settled confined colonies of the same morphological nature that were chosen from the cultured media. Every colony was contacted with a blazed wire-loop. Overnight hatching was done at rotating shaker at 37°C. The strain growth moved into a sanitized test tube containing 5 mL sterile saline H_2_O. The test tubes containing the bacterial suspension were vortex to be blended well consistently. At that point, the bacterial suspension was attuned with 0.5 barium sulfate turbidity norms. The attunement and evaluation of turbidity of inoculum tubes were detected by observing them visually with nude eye against a 0.5 barium sulfate turbidity equivalence stock with grey background and distinct blue lines in passable light. The attuned bacterial suspensions would be used as inoculum within fifteen minutes; if not, they cannot be used for analysis [36].

*(2) Disc Diffusion Method*. For the determination of the antibacterial property of aqueous and solvent extracts, the disc diffusion method was adopted with few modifications [37]. Inoculum of each bacterial culture to be tested with concentration of 10^6^ CFU/mL was spread on nutrient agar dishes with sterilized gauze saturated with the bacterial suspension. In this manner, discs with diameter of 9 mm were perforated into the agar medium and loaded up with concentration of 100 *μ*L (10 mg/mL of deionized H_2_O) of all plant extracts and antibiotic disc and permitted to diffuse at ambient temperature for two hours. The nutrient agar plates were then incubated in the upstanding situation at 37°C for overnight. Ciprofloxacin (250 mg), the standard antibiotic, was utilized as the positive control. After incubation of 24 hours, the diameters of inhibition zones were recorded in mm with the help of Caliber, and all plant samples were verified in triplicates. The data was mentioned as mean ± SD.

### 2.8. Antifungal Activity

Two fungal strains including *Aspergillus niger (A. niger)* (ATC 1688) and *Aspergillus flavus* (*A. flavus)* (IL 152) were experienced for antifungal efficiency of root, stem, bark, leaves, unripe fruit, and ripe fruit extracts of test plant. These two fungal strains were attained from the Department of Botany, Govt. College University, Faisalabad, Pakistan, and these strains were sustained at 4°C on potato dextrose agar for further experiment. An inoculum of fungal strains of *A. niger* and *A. flavus* was suspended in 5 mL potato dextrose agar and hatched at 37°C for 48 hours. The antifungal potency was evaluated by the disc diffusion method [37] with some modifications. In this method, the inoculum was spread evenly over medium of potato dextrose agar with sterilized glass diffuser. Small round paper discs with diameter of 9 mm were perforated into the agar medium and loaded up with concentration of 100 *μ*L (10 mg/mL of deionized H_2_O) of each plant extract and antibiotic standard and permitted to diffuse in medium at ambient temperature for absorption of plant extracts and then kept in the incubator for 24 to 48 hrs.at 37°C. The antifungal activity was assessed by estimating the diameter of zone of inhibition by using Caliber. Novidate (500 mg), antibiotic standard, was utilized as positive control. Triplicate values were measured for all tested extracts.

#### 2.8.1. Minimum Inhibitory Concentration (MIC)

MIC is the lowermost concentration observed in maintaining the capability of inoculums. Based on the primer screening, ethanol and methanol removes that uncovered intense antimicrobial action were additionally tried to select the minimum inhibitory concentration (MIC) for each bacterial example (Table 2). The serial dilution method was used to find the MIC of plant extracts against both gram-positive and gram-negative bacteria. To obtain a stock solution by following the disc diffusion method [38], the sample extract was dispersed in 1 mL of deionized H_2_O. Afterward, it was diluted to 10 folds by the sequential dilution method in which 9.5 mL of H_2_O and 0.5 mL of sample were added in the test tube. Then, 0.5 mL was taken from this test tube and added into another test tube having 9.5 mL water. This process was repeated 10 times, and then solutions of 10%, 40%, 70%, and 90% were prepared for the antibacterial and antifungal tests.

### 2.9. Statistical Analysis

Values were accounted for as the mean ± S.D. of three different experiments. For analysis and organization of resulting data, descriptive statistics were applied. For the analysis of data, two-route ANOVA with replications was used. Significance of information was tried by examination of fluctuation and Turkey (HSD) test at *p* < 0.05 and where appropriate at *p* < 0.01 utilizing XL-STAT programming. Information was additionally exposed to principal component analysis utilizing PC programming Microsoft Excel alongside XLSTAT Version 2012.1.02, Copyright Add in soft 1995-2012 (http://www.xlstat.com).

## 3. Results and Discussions

### 3.1. Extraction Yield

The yield percentage shows the extract amount gained from the extraction method stated in gram (g) of extracts found from per 100 gram (g) of crude plant powder and shown in Table 3.

The maximum yield was achieved in methanol bark extract (18.8 g/50 g of crude powder) followed by ethanol bark extract (14.2 g/50 g) while the petroleum ether extract indicated the lowest yield (4.2 g/50 g of crude powder) amongst all plant solvent extracts. In present investigations, polarity dependent intensification in extraction yield of different solvent extracts may be ascribed to high affinity for antioxidant components towards more polar solvents as compare to nonpolar solvents. The greater yield in polar solvent (methanol and ethanol) extracts specifies the polar nature of most of the phytochemicals in *D. malabarica* plant extracts. The lower yield in nonpolar solvent extracts showed a lower amount of nonpolar compounds in *D. malabarica* and the following order: methanol > ethanol > water > ethylacetate > dichloromethane > petroleum ether extract (Table 3). So, these outcomes were found in partial agreement with those investigated earlier [39].

The obtained results indicated that polar solvents as methanol followed by ethanol could be the best and effective solvents to extract more phytochemicals as compared to petroleum ether, ethyl acetate, and dichloromethane and can act as free radical scavengers because phytochemical contents determine their antioxidant capacity. On the other hand, the highest yield obtained in bark extracts amongst other plant parts demonstrated that *D. malabarica* bark extracts are a superb source of phytochemicals. Accordingly, the methanol, most polar solvent after water as testified formerly due to exhibiting more extraction yield, might be responsible to reflect more presence of phytochemicals and effective solvent in evaluation of potent biological activities [40].

### 3.2. Qualitative Phytochemical Screening

*D. malabarica* root, stem, bark, leaves, unripe fruit, and ripe fruit extracts were prepared by using more polar solvents (i.e., water, methanol, and ethanol) and less polar solvents (i.e., ethyl acetate, dichloro methane, and petroleum ether). The phytochemical constituents such as tannins, saponins, terpenoids, and flavonoids, of all tested plant extracts, were determined by qualitative analysis while total phenol content (TPC) and total flavonoid content (TFC) were determined through quantitative analysis.

Qualitative phytochemical screening of different extracts of *D. malabarica* in different solvents affirmed the presence of terpenoids, tannins, saponins, and flavonoids as shown in Table 4. The phytochemical screening (Table 3) revealed tannins with the affirmation of blue-black color while saponins and flavonoids confirmed their presence by the froth formation and yellow color, respectively. The subsequent appearance of the reddish-brown color on the inner face indicated the presence of terpenoids in the plant. In our study, the phytochemical screening showed that different tested plant organs of *D. malabarica* are a good source of flavonoids which could be supported by previous report [41] which displayed efficacy of each phytochemical for various biological action, like flavonoids show a vital role in antioxidant capability. Likewise, other phytochemicals as terpenoids, saponins, and tannins also demonstrate the potential of plants towards antibacterial and antifungal activities while recent studies also provide an overview of saponins about antiobesity healing prospective of saponins separated from therapeutic plants [42]. Plants enclose phytochemical compounds that were extracted and mostly utilized to heal some sorts of health-linked ailments and also exploited in production of food supplement and other nutrients. Each phytochemical reflects innovative biological actions that may possibly enhance the probabilities in detecting new antibiotic components against microbes [43]. In general, phytochemicals have substantial antioxidants, antimicrobial, anti-inflammatory, antiviral and resistant function, purification, and other functions of cell [44]. So, our present investigations were in agreement with earlier report which demonstrated the presence of phytochemicals like flavonoids, phenolic, saponins, alkaloids, sterols, tannins, and triterpenoids in *D. malabarica* plant extracts. Moreover, strong *in vitro* antioxidant potential due to the abundance of phytochemicals as terpenoids and flavonoids in ethanol extract of *D. malabarica* bark has been reported. The phytochemicals are being explored on priority for nutritive and herbal medicinal products [45]. Accordingly, in our current study, all tested parts of *D. malabarica* especially bark extract due to the greatest presence of tannins, saponins, terpenoids, and flavonoids could be a new addition in the production of phytomedicines and may be used for above reported similar trials as well as methanol may be considered as the optimal solvent to obtain high content of phytochemicals.

Accordingly, for the further quantitative determination of TPC and TFC and evaluation of biological activities, only methanol and ethanol solvent extracts of *D. malabarica* were screened due to reflecting their highest extraction yield and greatest presence of phytochemicals only in polar solvent as methanol and ethanol than nonpolar solvents.

### 3.3. Quantitative Phytochemical Analysis

#### 3.3.1. Determination of TPC and TFC

Total phenolic and flavonoid content of *D. malabarica* plant extracts has been expressed as *μ*g EAG/mg and *μ*g EQ/mg, respectively. All tested plant extracts demonstrated certain amount of TPC and TFC. The maximum level of phenols (602 ± 0.001 *μ*g EAG/mg of extract) and flavonoids (455 ± 0.6 *μ*g EQ/mg of extract**)** was found in the methanol bark extract, and minimum was in ethanol stem extract (Table 2). This concludes the fact that most of the phenolic, flavonoids in all tested parts of plant were taken out by more polar solvent, methanol than ethanol as stated in previous report [46]. So, highest values of TPC and TFC in methanol indicated that most of the phenolic compounds found in *D. malabarica* might be polar in nature. On the other hand, the bark extract exhibited the highest amount of TPC and TFC amongst other tested extracts of *D. malabarica*. The results showed that methanol bark extract amongst other extracts could be more effective and fight against free radicals because antioxidant capability is determined by phytochemical contents as it was found in partial agreement of earlier report [47, 48] which demonstrated that antioxidant potency of methanol bark extract of *D. malabarica* might be due to the greatest presence of well-known natural antioxidants such as polyphenol, tannins, and flavonoids. Of all phytochemicals, polyphenols are greatly identified as an antioxidant anti-inflammatory, antiviral, and antimicrobial agents [49]. TFC and TPC are also reported to prevent DNA from oxidative stress including inhibition of tumor cell growth and exhibit antimicrobial and anti-inflammatory activities. The previous investigations also revealed the role of phenolic compounds as a great source of antioxidant activity in *D. malabarica* [50], and the flavonoids are polyphenols which play a vital role in antibiotics action [51] because only flavonoids are involved in making complexes with microbial proteins, cell wall, and many other components that are responsible for biological function.

### 3.4. Biological Activities

#### 3.4.1. Anti-Inflammatory Activity (Inhibition of Albumin Denaturation)

**Figure 2:**
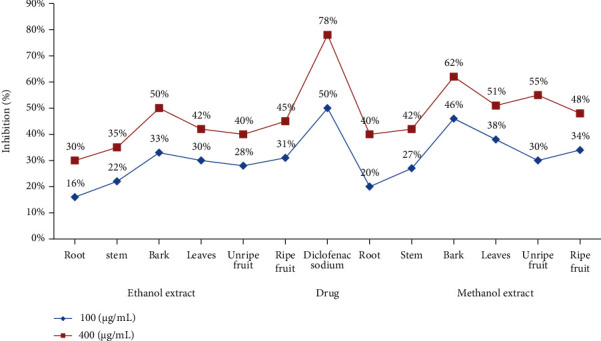
Albumin denaturation inhibitory activity of different *D. malabarica* extracts.

Protein denaturation is a method through which protein structure is demolished due to the presence of external stress, oxidative stress, other compounds, or heat; so, it becomes responsible to fail their biological activity. Hence, the denaturation of tissue proteins due to oxidative damage is documented as a symbol of inflammation. Here, the *in vitro* anti-inflammatory activity of *D. malabarica* extracts was evaluated for hindrance against protein denaturation.  Figure 2 shows the inhibitory influence of different *D. malabarica* extracts on protein denaturation. *D. malabarica* methanol bark extract amongst other extracts exposed significantly 62% greater protein protection near around diclofenac sodium (78%), a standard anti-inflammatory drug, at a concentration of 400 *μ*g/mL which may be attributed due to the rich diversity of phytochemical constituents (tannins, phenols, flavonoids, saponins and terpenoids) in greater amounts as well as the solvent type used to extract bioactive components completely found within *D. malabarica* plant as reported formerly [52]. The three triterpenoid compounds such as betulin, betulic acid, and ursolic acid isolated from *D. malabarica* have been reported to exert pronounced anti-inflammatory activity [5]. Similarly, another reported significant anti-inflammatory activity in *D. malabrica* bark extract [53] encouraged our present findings.

#### 3.4.2. Anticancer Activity

**Figure 3:**
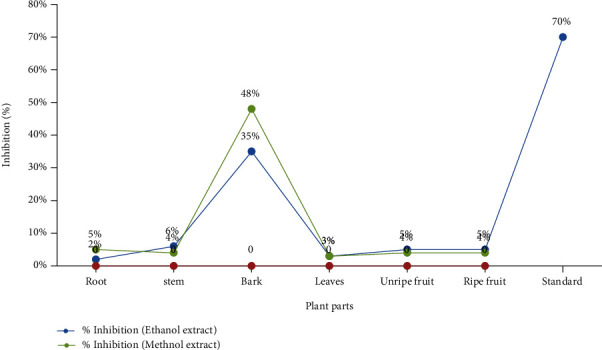
Anticancer activity of different *D. malabarica* extracts (30 *μ*g/mL).

The cytotoxic results of ethanol and methanol extracts of *D. malabarica* against the HeLa cell line are shown in Figure 3. All tested extracts presented a bit anticancer activity but methanol bark extract of *D. malabarica* showed a potent anticancer effect (48%) while doxorubicin (70%) was used as a standard anticancer drug. Although resulted from anticancer activity in *D. malabarica* bark extract was quite less than standard but found not to be inactive even at very low concentration (100 *μ*g/mL) which could be the result of remarkable polyphenols including total flavonoids and tannins consisting of antioxidant properties that may act as anticancer agents as these phytochemicals were reported earlier as anticancer compounds [34] and may be the substitute of conventional chemotherapy or however reduce its side effects, our finding may be in agreement with earlier investigations which revealed an extensive optimistic connection between diverse secondary metabolites like flavonoids, phenolics, tannins, and saponins with antioxidant and anticancer potency. It is reported that a person's diet involving polyphenols being natural antioxidants can improve health and reduce the risk of cancers [54].

#### 3.4.3. Antidiabetic Activity (*α*-Amylase Inhibition)

**Figure 4:**
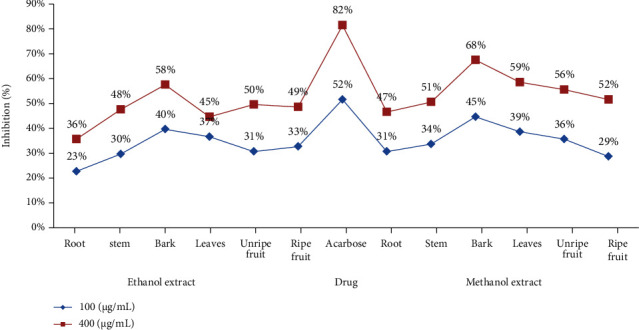
*α*-Amylase inhibitory activity of different *D. malabarica* extracts.

The methanol bark extract of *D. malabarica* followed by ethanol extract at concentrations of 100 and 400 *μ*g/mL revealed substantial antidiabetic activity. The methanolic bark amongst other extracts exhibited 68% *α*-amylase inhibition (Figure 4) which was not very close to acarbose (82%), a standard antidiabetic drug but reflected significant antidiabetic activity even at a very low concentration 400 *μ*g/mL. This antidiabetic potential of bark extract may be attributed to the presence of flavonoids and terpenoids as reported by Kavatagimath and Jalalpure [55] reinforcing our findings. Previously, methanolic leaf extract was reported to exhibit good antihyperglycemic activity in glucose tolerance tests and alloxan-induced diabetic rats [55]. Consequently, the methanol bark extract of *D. malabarica* might be used as a potential *in vivo* antidiabetic agent. The *α*-amylase inhibitory activity in methanol extract is most likely to be due to polar compounds and is worth investigating further and isolating pure active compounds [56].

#### 3.4.4. Antibacterial Activity

In the course of evaluation for antibacterial activity by the disc diffusion method, all the *D. malabarica* extracts presented different degrees of antibacterial activity (9.50 ± 1.2 − 19.25 ± 1.9) mm against *E.coli*, the gram-negative bacteria, and (9.75 ± 0.9 − 30.25 ± 0.9) mm against *streptococcus*, the gram-positive bacteria (Table 2). The methanol bark extract displayed the strongest zone of inhibition against *streptococcus* (30.25 mm) while ethanol unripe fruit extract exhibited (19.25 mm) against *E.coli* amongst all extracts while the zone of inhibition reflected by standard ciprofloxacin 250 mg was 45.5 mm. Antibacterial action was discovered to be more articulated against gram-positive microscopic organisms contrasted with gram-negative. This discloses itself to the earlier investigations demonstrating that plant extracts have greater ability against gram positive microorganisms than gram-negative [57]. In this overall antimicrobial study, the methanolic extract exhibited strong activity than petroleum ether extract as investigated previously [58]. Methanol showed more reliable and protuberant antibacterial activity as compared to ethanol extracts (Table 5). The minimal antibacterial potency in ethanol extracts might be due to presence of lower concentration of antibacterial components in these plant extracts [39]. A number of studies have been executed that ascribe the phenolics to kill microbes. It can be ascribed from the fact that high content of phytochemicals present in plant extracts might be reflected as the source for antimicrobial potency as appealed earlier that plants rich in tannin and saponin have intense antimicrobial action [59]. Antibacterial activity of medicinal plants was reported by several investigations [60] which supported our research finding. The antibacterial activity has been reported earlier in *D. malabarica* leaves against various diseases [5] while our study revealed significant antibacterial activity in the methanol bark extract which may be attributed for similar trials. The MIC values of different parts of *D. malabarica* are shown in Table 5. MIC of all extracts was found in 90%, 70%, 40%, and 10% concentrated sample against *E. coli* and *Streptococcus.* The inhibitory zone was shown at 90% while no inhibition was found at a concentration of 10% which may be due to absence of antibacterial components at lower concentratins in plant extracts. Accordingly, our current study is supported by earlier literature conclusions that antibacterial activities are directly related to increasing the concentration (%) of extracts [61]. Although various classes of phytochemical constituents were described showing antimicrobial capabilities, however, they are not recognized as tonic by medical community. In current finding, worthy antibacterial action was displayed by methanol bark extract against the gram-positive bacteria which is as per the previous investigation which revealed the methanol extract to have the most extreme flavonoid and phenolic levels and showing more articulated antibacterial activity contrasted with other solvent extracts [62].

#### 3.4.5. Antifungal Activity

The current study assesses the antifungal activity of *D.malabrica* extracts. Ethanol and methanol extracts of *D. malabarica* plant parts were screened for their antifungal activities against fungal strains *A. niger* and *A. flavus* (Table 6). Here, methanol bark extract showed the highest 18.25 mm zone of inhibition against *A. flavus* and 17.50 mm against *A. niger* (Table 7) which was found very close to novidate 500 mg (20.25 mm), the standard antifungal drug. The obtained results indicated that extracts of *D. malabarica* were found not to be inactive against *A. niger* and *A. flavus* even at low concentrations of 10 mg/mL and showed antifungal activity but methanolic bark extract amongst other extracts of *D.malabarica* comprises strong antifungal activity against microorganisms that may be due to the presence of tannins, saponins, flavonoids, terpenoids, and phenolic compounds in bark extract as indicated in the previous study that saponins might be attributed to fight fungal microbes [63]. The phytochemicals including flavonoids and phenolic compounds found in plants as secondary metabolites may be responsible of antimicrobial activity [64]. Accordingly, earlier reports supported our research finding regarding potent antifungal activity possessed by methanol bark extract *D. malabarica* [65]. This may be because of the used type of solvent for extraction as portrayed earlier that methanol is the more effective solvent for extraction of bioactive compounds particularly antimicrobial components from therapeutic plants when contrasted with different solvents as well as water [61].

## 4. Conclusions

*D. malabarica* bark extract attained from the methanol, a polar solvent, showed a significant measure of phytochemicals than less polar or nonpolar solvents. Methanol is the more effective solvent for extraction of bioactive compounds particularly antimicrobial components from therapeutic plants when contrasted with different solvents. In this way, it is predicted that in the future drugs emerging from these phytochemicals will be entity of a developing interest for infection and oxidative stress related illnesses. We believe that the recognizable proof of the potential medical advantages of phytochemicals might be a vital factor to give further bits of knowledge into the revelation of medications or effective nutrition. So, *D. malabarica* bark being rich in various phytochemical compounds with various healing tendency can be utilized as a potent source of natural antioxidants with other curative applications including antihepatotoxicity. *D. malabarica* bark might be screened further against different infection causing microorganisms and can be an expected source of biologically significant medication competitors.

## Figures and Tables

**Table 1:** Various extraction techniques with different optimization conditions for natural products.

Method	Solvent	Temperature	Time	Volume of solvents used
Maceration	Water, aqueous, nonaqueous solvents	Room temperature	Long	Large
Soxhlet extraction	Organic solvents	Under heat	Long	Moderate
Supercritical fluid extraction	Supercritical fluid (usually S-CO_2_), sometimes with modifier	Near room temperature	Short	None or small
Ultrasound-assisted extraction	Water, aqueous, and nonaqueous solvents	Room temperature or under heat	Short	Moderate
Microwave-assisted extraction	Water, aqueous, and nonaqueous solvents	Room temperature	Short	None or moderate
Reflux extraction	Aqueous and nonaqueous solvents	Under heat	Moderate	Moderate

**Table 2:** Estimation of TPC and TFC of *D. malabarica* extracts.

Extracts	Solvents	Total phenols (*μ*g EAG/mg)	Total flavonoids (*μ*g EQ/mg)
Root	Methanol	299 ± 0.004	285 ± 0.7
	Ethanol	246 ± 0.002	204 ± 0.8
Stem	Methanol	208 ± 0.005	196 ± 0.2
	Ethanol	191 ± 0.005	106 ± 0.1
Bark	Methanol	602 ± 0.001	455 ± 0.6
	Ethanol	534 ± 0.002	432 ± 0.5
Leaves	Methanol	440 ± 0.001	379 ± 1.2
	Ethanol	383 ± 0.001	368 ± 1.1
Unripe fruit	Methanol	467 ± 0.04	211 ± 1.2
	Ethanol	416 ± 0.07	220 ± 1.7
Ripe fruit	Methanol	450 ± 0.002	301 ± 1.5
	Ethanol	397 ± 0.08	237 ± 1.0

EAG: equivalent gallic acid; EQ: equivalent quercetin.

**Table 3:** Extraction yield (% w/w) of *D. malabarica* extracts prepared using different solvents.

Solvents	Extract	Powdered mass (g)	Extracted mass (g)	Yield (%)
Petroleum ether	Bark	50	2.1	4.2
Dichloro methane		50	2.3	4.6
Ethyl acetate		50	2.6	5.2
Ethanol		50	7.1	14.2
Methanol		50	8.9	17.8
Aqueous		50	2.3	4.6
Petroleum ether	Stem	50	1	2
Dichloro methane		50	1.2	2.4
Ethyl acetate		50	1.3	2.6
Ethanol		50	2.2	4.4
Methanol		50	3.3	6.6
Aqueous		50	1.4	2.8
Petroleum ether	Leave	50	1.3	2.6
Dichloro methane		50	1.5	3
Ethyl acetate		50	1.9	3.8
Ethanol		50	6.6	13.2
Methanol		50	6.9	13.8
Aqueous		50	1.9	3.8
Petroleum ether	Unripe fruit	50	1.3	2.6
Dichloromethane		50	1.5	3
Ethyl acetate		50	1.7	3.4
Ethanol		50	4.3	8.6
Methanol		50	4.9	9.8
Aqueous		50	2.2	4.4
Petroleum ether	Ripe fruit	50	1.4	2.8
Dichloro methane		50	1.6	3.2
Ethyl acetate		50	1.8	3.6
Ethanol		50	3.8	7.6
Methanol		50	4.4	8.8
Aqueous		50	2	4
Petroleum ether	Root	50	0.5	1
Dichloro methane		50	0.6	1.2
Ethyl acetate		50	0.9	1.9
Ethanol		50	3.4	6.8
Methanol		50	4.9	8.1
Aqueous		50	1.9	3.8

**Table 4:** Comprehensive view of phytochemical constituents in *D. malabarica.*

Solvents	Extract	Tannin	Saponin	Flavonoid	Terpenoid
Petroleum ether	Bark	+	+	+	+
Dichloro methane		+	+	+	+
Ethyl acetate		+	+	+	+
Ethanol		+	++	+	+
Methanol		++	++	++	++
Aqueous		+	+	+	+
Petroleum ether	Stem	+	+	+	—
Dichloro methane		+	+	—	—
Ethyl acetate		+	+	+	—
Ethanol		—	+	+	—
Methanol		+	—	+	—
Aqueous		+	+	—	—
Petroleum ether	Leave	+	—	+	+
Dichloro methane		+	+	+	+
Ethyl acetate		+	—	+	+
Ethanol		+	—	+	+
Methanol		+	—	+	+
Aqueous		+	—	+	+
Petroleum ether	Unripe fruit	+	+	+	+
Dichloro methane		+	+	+	+
Ethyl acetate		+	+	+	+
Ethanol		+	+	+	+
Methanol		+	+	+	+
Aqueous		+	+	+	+
Petroleum ether	Ripe fruit	—	+	+	+
Dichloro methane		—	—	+	—
Ethyl acetate		+	+	+	+
Ethanol		—	+	+	+
Methanol		+	+	++	++
Aqueous		+	—	—	+
Petroleum ether	Root	—	+	+	—
Dichloro methane		+	+	—	+
Ethyl acetate		—	+	+	—
Ethanol		+	+	+	+
Methanol		+	+	+	+
Aqueous		+	+	—	+

+: low color intensity, +++: high color intensity; -: absence of coloration.

**Table 5:** Antimicrobial activity of different *D. malabarica* solvent extracts.

Extracts	Solvents		^∗^ *Mean* ± *S*.*D*.	MIC			^∗^ *Mean* ± *S*.*D*.	MIC	
				Concentration	Mean			Concentration	Mean
Ciprofloxacin (250 mg)	Standard		39.50 ± 2.3				45.50 ± 0.9		
Root	Methanol	E. coli (gram-negative bacteria)	11.50 ± 2.0	90%	9.25	*Streptococcus* (gram-positive bacteria)	11.25 ± 0.9	90%	10.00
				70%	Nil			70%	Nil
				40%	Nil			40%	Nil
				10%	Nil			10%	Nil
	Ethanol		9.50 ± 1.2	90%	9.25		13.25 ± 0.9	90%	12.00
				70%	Nil			70%	9.00
				40%	Nil			40%	Nil
				10%	Nil			10%	Nil
Stem	Methanol		11.75 ± 1.7	90%	9.75		15.25 ± 1.2	90%	9.75
				70%	Nil			70%	Nil
				40%	Nil			40%	Nil
				10%	Nil			10%	Nil
	Ethanol		10.25 ± 1.5	90%	9.25		9.75 ± 0.9	90%	9.25
				70%	Nil			70%	Nil
				40%	Nil			40%	Nil
				10%	Nil			10%	Nil
Bark	Methanol		12.00 ± 1.8	90%	9.50		30.25 ± 0.9	90%	12.75
				70%	Nil			70%	9.50
				40%	Nil			40%	Nil
								10%	Nil
				10%	Nil				
	Ethanol		11.50 ± 2.0	90%	9.25		15.75 ± 1.7	90%	14.00
				70%	Nil			70%	10.75
				40%	Nil			40%	Nil
				10%	Nil			10%	Nil
Leaves	Methanol		11.75 ± 1.5	90%	9.5		17.00 ± 1.4	90%	11.75
				70%	Nil			70%	Nil
				40%	Nil			40%	Nil
				10%	Nil			10%	Nil
	Ethanol		11.5 ± 1.9	90%	9.25		15.75 ± 0.9	90%	12.50
				70%	Nil			70%	9.25
				40%	Nil			40%	Nil
				10%	Nil			10%	Nil
Unripe fruit	Methanol		10.50 ± 1.5	90%	14.25		18.25 ± 1.7	90%	12.75
				70%	12.25			70%	9.25
				40%	9.75			40%	Nil
				10%	Nil			10%	Nil
	Ethanol		19.25 ± 1.9	90%	9.75		14.25 ± 0.9	90%	9.50
				70%	Nil			70%	Nil
				40%	Nil			40%	Nil
				10%	Nil			10%	Nil
Ripe fruit	Methanol		13.25 ± 1.2	90%	10.5		14.25 ± 1.2	90%	9.75
				70%	Nil			70%	Nil
				40%	Nil			40%	Nil
				10%	Nil			10%	Nil
	Ethanol		13.50 ± 1.9	90%	10.25		10.25 ± 1.5	90%	9.25
				70%	9.25			70%	Nil
				40%	Nil			40%	Nil
				10%	Nil			10%	Nil

^∗^Values are mean of duplicates of zone of inhibition (mm**).**

**Table 6:** Antifungal potential of different *D. malabarica* solvent extracts.

Extracts	Solvents	*A. niger*	^∗^ *Mean* ± *S*.*D*	*A. flavus*	^∗^ *Mean* ± *S*.*D*
Novidate (500 mg)	Standard		20.25 ± 0.5		20.50 ± 0.5
Root	Methanol		11.75 ± 0.2		9.75 ± 0.2
	Ethanol		10.50 ± 0.1		9.25 ± 0.5
Stem	Methanol		9.50 ± 0.5		10.00 ± 0.8
	Ethanol		12.75 ± 0.5		13.00 ± 1.8
Bark	Methanol		17.50 ± 0.3		18.25 ± 0.2
	Ethanol		12.25 ± 0.7		15.00 ± 0.8
Leaves	Methanol		10.00 ± 0.8		12.25 ± 0.2
	Ethanol		14.75 ± 0.5		15.00 ± 0.1
Unripe fruit	Methanol		13.50 ± 0.5		15.25 ± 1.7
	Ethanol		10.00 ± 0.8		13.75 ± 0.7
Ripe fruit	Methanol		11.75 ± 0.7		12.75 ± 0.2
	Ethanol		14.25 ± 0.2		16.25 ± 0.1

^∗^Values are mean of duplicates of zone of inhibition (mm).

**Table 7:** Conclusive view of biological activities of *D.malabarica* extracts.

Extracts	Solvents	Biological activities				
		Anti-inflammatory	Anticancer	Antidiabetic	Antibacterial	Antifungal
Root	Methanol	++	++	++	++	++
	Ethanol	+	+	+	+	+
Stem	Methanol	++	++	++	++	+
	Ethanol	+	+	+	+	++
Bark	Methanol	++++	++++	++++	++++	++++
	Ethanol	++	++	++	++	++
Leaves	Methanol	++	+	++	++	+
	Ethanol	+	+	+	+	++
Unripe fruit	Methanol	++	+	++	+	+
	Ethanol	+	+	+	++	++
Ripe fruit	Methanol	++	+	++	++	+
	Ethanol	+	+	+	+	++

(+) and (+++) signs indicate the low intensity and high intensity of biological activities, respectively.

## Data Availability

All the data relevant to this study is mentioned in the manuscript. There is no supplementary data.
